# Low-Complexity 3D InISAR Imaging Using a Compressive Hardware Device and a Single Receiver

**DOI:** 10.3390/s22155870

**Published:** 2022-08-05

**Authors:** Mor Diama Lo, Matthieu Davy, Laurent Ferro-Famil

**Affiliations:** 1 IETR, University of Rennes 1, 35000 Rennes, France; 2 ISAE-SUPAERO, University of Toulouse, 31055 Toulouse, France; 3 CESBIO, University of Toulouse, CNES/CNRS/INRAE/IRD/UPS, 31401 Toulouse, France

**Keywords:** ISAR, interferometric ISAR, radar, compressive device, computational imaging

## Abstract

An Interferometric Inverse SAR system is able to perform 3D imaging of non-cooperative targets by measuring their responses over time and through several receiving antennas. Phase differences between signals acquired with a spatial diversity in vertical or horizontal directions are used to localize moving scatterers in 3D. The use of several receiving channels generally results into a costly and complex hardware solution, and this paper proposes performing this multichannel acquisition using a single receiver and a hardware compressive device, based on a chaotic cavity which simultaneously multiplexes in the spectral domain signals acquired over different antennas. The radar responses of the scene are encoded in the spectral domain onto the single output of a leaky chaotic cavity, and can be retrieved by solving an inverse problem involving the random transfer matrix of the cavity. The applicability of this compressed sensing approach for the 3D imaging of a non-cooperative target using low-complexity hardware is demonstrated using both simulations and measurements. This study opens up new perspectives to reduce the hardware complexity of high-resolution ISAR systems.

## 1. Introduction

Inverse Synthetic Aperture Radar (ISAR) is a technique used for many applications related to target classification and recognition, due to its ability to provide high-resolution images of a scene. In its simplest form, two-dimensional (2D) ISAR imaging may be associated with the filtered projection of three-dimensional target reflectivity onto the Image Projection Plane(IPP) [1,2]. These images are generated by estimating the typically unknown motion of the target relative to the observing the radar [1,3] or using specific properties of the radar responses of artificial objects [4]. The interpretation of 2D conventional ISAR images is, however, difficult most of the time [1,2,5], and three-dimensional ISAR images with detailed geometric feature of the scatterers, such as their lengths, widths and heights, are necessary for target recognition and classification [5].

Several techniques have been proposed to form 3D ISAR images. Firstly, a time-domain technique approach [6,7] using a single sensor based on 3D matched filtering [8,9] for 3D reconstruction of non-cooperative targets. This approach, which uses a sequence of ISAR images [10,11,12,13], requires a long observation time and accurate scattering point extraction and scattering point trace matrix decomposition. The corresponding algorithm, therefore, has considerable computational complexity. Another approach is based on interferometric principles and makes use of multiple receivers [14,15,16,17]. This spatial diversity provides height information which makes it possible to separate scatterers with the same range–Doppler values. More generally, 3D-ISAR interferometric images are formed using a two-dimensional antenna array. In [5,18], a L-shaped array configuration was proposed to estimate the height of the target main scattering centers from the joint phases difference between ISAR images computed at different antenna locations. This technique can be implemented with a short observation time, without the need for a scatterer tracking algorithm. However, its accuracy depends on the relative distance between the target dimensions and receiver baselines. 3D InISAR was demonstrated experimentally for both complex land [19] and maritime targets [20,21]. Finally, the last approach relies on a 3D bistatic ISAR imaging technique to estimate scattering centers in 3D cartesian space [22,23].

In interferometric approaches, the signals must, however, be recorded on a synchronized antenna array which increases the price and complexity of the system. Here, we combine 3D Interferometry Inverse Synthetic Aperture Radar and computational imaging to obtain 3D images of a scene from a single receiving channel. The principle of computational imaging techniques in the microwave range is to connect the array of receiving antennas to a dispersive component with a high quality (Q) factor and encode the signals onto a single spectrum. The reverberation within the component provides a space/frequency transfer matrix between the inputs and the output signals, which is random given that the spacing between two frequencies is larger than the spectral correlation length of the device [24,25]. The spectral correlation length is typically equal to 1/τ, where τ is the temporal decay factor of transmitted signals. Using that, the random transfer functions of the cavity are nearly orthogonal; the incoming signals can be reconstructed numerically by solving an inverse problem with deconvolution techniques such as an adaptative filter or matrix inversion [26]. This technique has the advantage of using a cheap component such as a photonic crystal [27], a multiple scattering medium [28,29] or a chaotic cavity [24,25,30,31,32,33].

In this article, we demonstrate numerically and experimentally that InISAR techniques can be combined with the use of chaotic cavity as a compressive sensing device to obtain high-resolution 3D images of a moving target. This paper is organized as follows: we first present the compressive sensing device and our methodology to solve the inverse problem in Section 2. After detailing the geometry of the InISAR system in Section 3 and the InISAR algorithm in Section 4, the Section 5 and Section 6 are devoted to numerical simulations and experimental results validating our approach.

## 2. Compressive Sensing Device

### 2.1. Device Description

**Figure 1:**
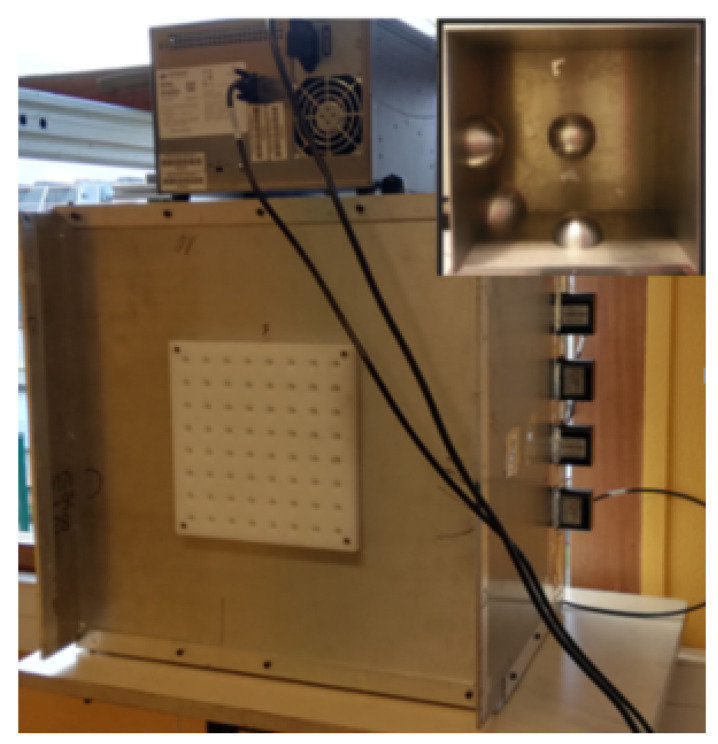
Photograph of the chaotic cavity. Four ports can be seen on the right hand wall of the cavity. The interior of the cavity, containing four hemispheres, is shown in the inset.

**Figure 2:**
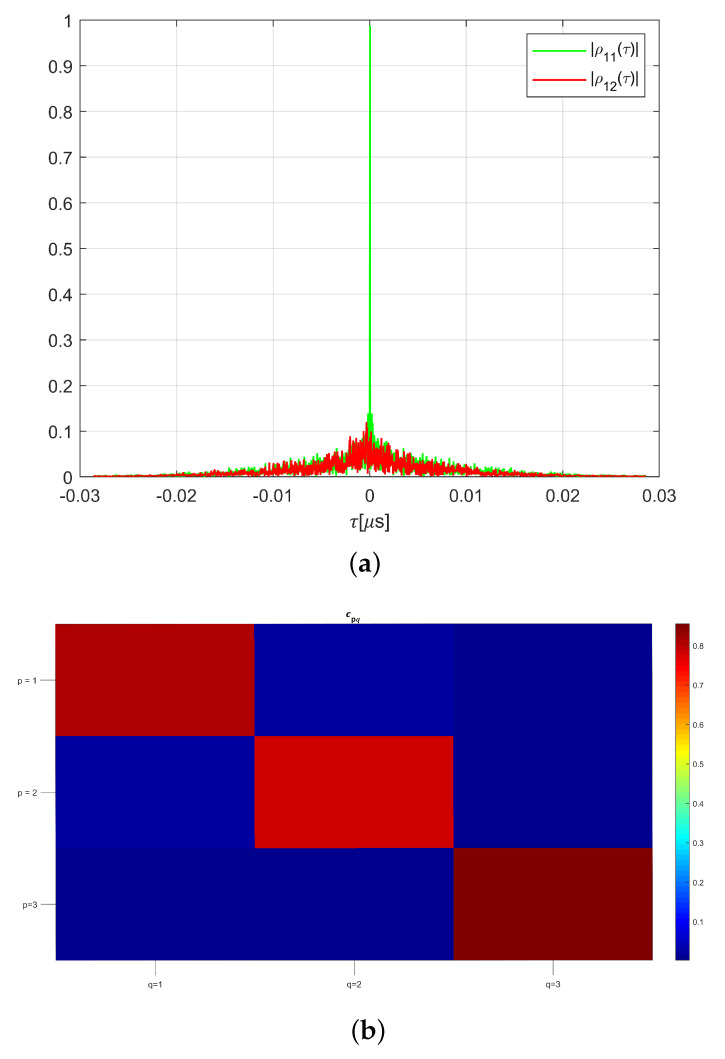
Features of the transfer functions of the chaotic cavity. (**a**) Amplitude of time—domain correlation functions (2); (**b**) Spectral transmission coefficients (3).

A *N* to 1 passive multiplexer, operating over a frequency band centered around $f_{0}=6$ GHz, and consisting of a metallic chaotic cavity manufactured at the IETR, can be seen in Figure 1). The cavity has a volume of 0.075 m^3^ and its dimensions, 50 cm × 50 cm × 30 cm, are all much larger than the carrier signal wavelength. The cavity is made fully chaotic by placing three metallic hemispheres on the walls, as well as a deformed corner, shown in Figure 1. Four coaxial to waveguide transitions are connected to the cavity through a wall in order to multiplex N=3 input signals, $s_{n}\left(\tau \right)$ with *n* = 1, 2 or 3, into a composite compressed signal y(τ), accessible at a fourth port on the wall, and measured in the spectral domain by a Vector Network Analyzer (VNA). Multiple scattering of waves occurring within this enclosure provides large spectral diversity that may be used to retrieve the input signals, $s_{n}\left(\tau \right)$ using the measured scalar information, y(τ), and the known chaotic impulse responses, $s_{n}\left(\tau \right)$, relating the inputs of the cavity to its output. The quality of the reconstruction is conditioned the number of degrees of freedom, or independent components that may be addressed over the measured spectral domain, given by [24,25]

(1) $$N_{f}=B\tau_{r}$$

where $\tau_{r}$ is the typical reverberation time of the cavity, i.e., the spread of its impulse response, approximately equal to 80 ns with the considered device, and *B* is the width of the measured spectral domain, equal to 2 GHz in this experiment. The configuration adopted in this work yields a high quality factor $Q=2\pi f_{0}\tau_{r}\approx 3000$ [24]. This leads to a minimum number of frequencies to code the information on all modes of the box $N_{f}\geq 1600$. Hence, we choose $N_{f}=2001$ spectral coordinates for a characterization of the transfer functions of the box. The cross-correlation between the impulse responses of ports *p* and *q* is formulated as

(2) $$\rho_{pq}\left(\tau \right)=c_{p}\left(\tau \right)⊗c_{q}^{*}\left(-\tau \right)$$

with ⊗ as the convolution operator, and where the individual impulse responses are normalized so that $\rho_{pp}\left(0\right)=1,\;\forall p$. The amplitude of the correlation functions $|\rho_{11}|$ and $|\rho_{12}|$, displayed in Figure 2a, shows a small degree of correlation between these two transfer functions and a rather narrow auto-correlation pattern. The orthogonality of the system transfer functions can be generally observed by calculating the matrix of transmission coefficients $c_{pq}$ in the frequency domain as

(3) $$c_{pq}=\sum_{j=1}^{N_{f}}\left|c_{p}\left(f_{j}\right)c_{q}^{*}\left(f_{j}\right)\right|/N_{f}$$

The matrix of correlation coefficients between the three transfer functions is close to diagonal, as shown in Figure 2b. A completely diagonal matrix would provide perfect reconstruction of incoming signals. However, the quality of the reconstruction of the inputs waveform is limited due to the finite value of $N_{f}$.

### 2.2. Vector Signal Retrieval Principle

**Figure 3:**
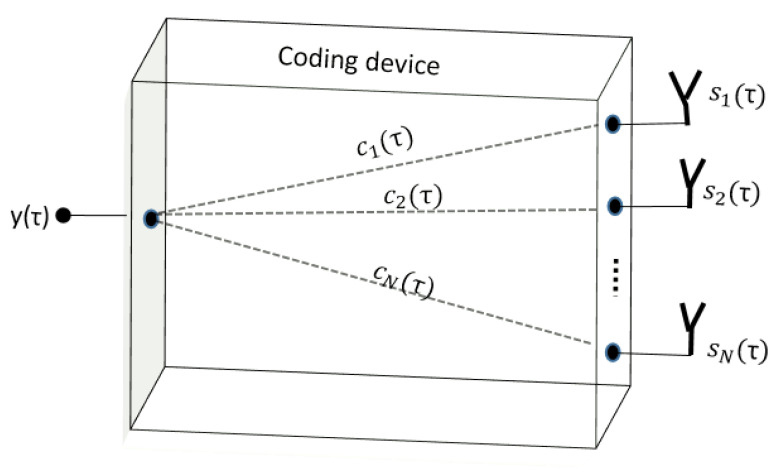
Synopsis of the hardware compressive coding device used in this study.

As illustrated in Figure 3, the output time-domain signal recorded for a N×1 passive coding device may be written as

(4) $$y\left(\tau \right)=\sum_{n=1}^{N}c_{n}\left(\tau \right)⊗s_{n}\left(\tau \right)+v\left(\tau \right),$$

where v(τ) stands for the acquisition noise. In the spectral domain, (4) simplifies to

(5) $$y=\sum_{n=1}^{N}c_{n}⊙s_{n}+v,$$

where ⊙ represents the elements-wise product, and y is defined as $y={\left[\begin{array}{c} y\left(f_{1}\right),\ldots ,y\left(f_{M}\right) \end{array}\right]}^{T}$ where y(f) stands for the Fourier transform of y(τ), and $f_{1},\ldots ,f_{M}$ represent spectral coordinates at which the received signal is measured. The expression of the measured signal in (5) may be represented using a linear transformation

(6) y=Cs+v,

with $C=\left[\begin{array}{c} C_{1},\ldots ,C_{N} \end{array}\right]\in C^{M\times MN}$, the cavity channel transfer matrix, $C_{n}=\mathrm{diag}\left(c_{n}\right)\in C^{M\times M}$, the transfer matrix of a single channel, and $s={\left[\begin{array}{c} s_{1}^{T},\ldots ,s_{N}^{T} \end{array}\right]}^{T}\in C^{MN}$, the input vector signal.

Among the the wide variety of inversion approaches which could be applied to retrieve s from y, quadratically regularized Linear Least Squares Estimation techniques are particularly interesting, as they can be implemented in the form of a linear transformation, and related to physical interpretations. The corresponding criterion to be minimized is given by

(7) $$J\left(s\right)={∥y-Cs∥}^{2}+\lambda \;{∥\Gamma s∥}^{2}$$

whose solution is $\hat{s}=Qy$ with

(8) $$Q={\left(C^{H}C+\lambda \;\Gamma^{H}\Gamma \right)}^{-1}C^{H}$$

where λ is a user-defined regularization weight and Γ is an arbitrary matrix, whose structure may be used to confer specific properties to the estimated signal. In particular, λ=0 corresponds to the non-regularized LLSE, whereas λ>0 leads to the so-called Tikhonov regularization, known to improve the conditioning of the inverse problem, which operates uniformly over the signal space if $\Gamma^{H}\Gamma =I$. For large λ values, and $\Gamma^{H}\Gamma =I$ the inversion approach is equivalent to the matched filter solution, with $Q\propto C^{H}$, whose features may be appreciated from the cross-correlation plots given in Figure 2, and which provides optimal signal-to-noise ratio characteristics after reconstruction.

## 3. Geometry and Signal Model

The InISAR system used in this study consists of a transmitting antenna ($P_{T}$), three receiving antennas ($P_{RC},P_{RH},P_{RV}$) and a chaotic box. The receiving antennas lie on both a horizontal and a vertical baseline with a L-shape configuration, as originally proposed in [5,18]. The receiving channels are connected to the inputs ports of the coding device and a single output signal is measured. The radar system is pointed at the scene with a vertical orientation β and a horizontal orientation α. The corresponding squint mode induces a geometrical distortion [34] of the synthesized InISAR images.

**Figure 4:**
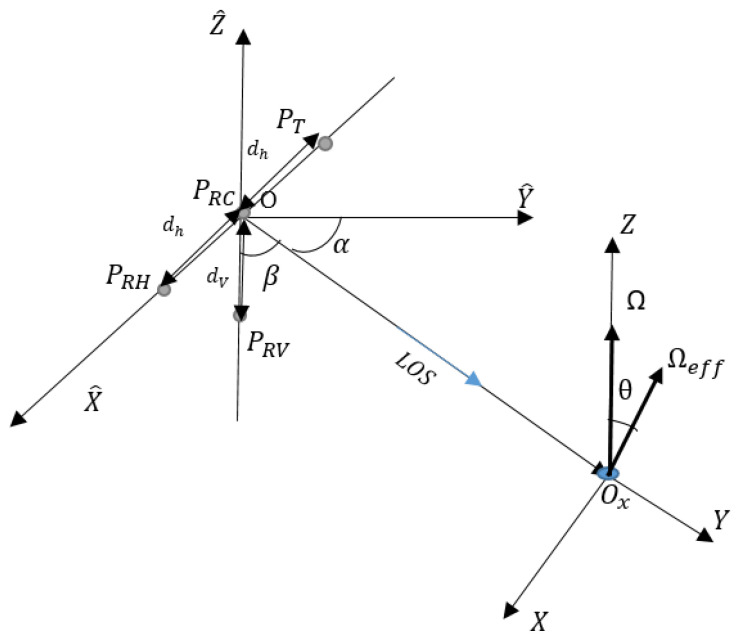
InISAR acquisition geometry.

The InISAR geometry is represented in Figure 4. The reference system $T_{0}=\left\{\hat{X},\hat{Y},\hat{Z}\right\}$ is expressed at the radar antenna array, whereas the reference system $T_{1}=\left\{X,Y,Z\right\}$ is centred on the centre of rotation of the target at t=0. The axis $\hat{Y}$ defines the ground range, while $\hat{X}$ and $\hat{Z}$ correspond to the horizontal and vertical baseline directions, respectively.

The target is assumed to be a rigid body consisting of $N_{t}$ point-like scatterers. The *Y*-axis is chosen to be the LOS direction. The projection of the rotation vector onto the plan orthogonal to *Y* defines the effective rotational vector $\Omega_{eff}\left(t\right)$. According to [35], the axis *X* and *Y* define the imaging plane, (*Y* corresponds to the range and *X* to the cross-range). The axis *Z* is tilted by an angle θ with respect to the effective rotation vector.

The antenna located at the position $P_{T}$ transmits a wide-band pulse. The signal back-scattered to the nth receiving antenna for a single point scatterer of reflectivity $a_{n}$, with time-varying position vector in the $T_{XYZ}$ frame denoted as x(t)=[x(t),y(t),z(t)] can be written as

(9) $$\begin{array}{c} \begin{array}{c} S_{n}\left(f,t\right)\approx a_{n}\exp\left(-j\frac{4\pi f}{c}d_{0_{n}}\left(t\right)\right)\\ \exp\left(-j\frac{4\pi f}{c}\left[x\;\cdot \;\left(i_{P_{T}}+i_{P_{n}}\right)\right]\right)W\left(f,t\right) \end{array} \end{array}$$

where

(10) $$W\left(f,t\right)=\mathrm{rect}\left(\frac{t}{T_{obs}}\right)\;\cdot \;\mathrm{rect}\left(\frac{f-f_{o}}{B}\right)$$

delimitates the frequency and the time domains over which signals are measured, with $T_{obs}$ as the observation time, $f_{0}$ as the carrier frequency, and *B* as the signal bandwidth. The distance $d_{0_{n}}\left(t\right)=\left(d_{P_{T}}\left(t\right)+d_{P_{Rn}}\left(t\right)\right)/2$ accounts for the distances between $P_{T}$ and the target center $O_{x}$ and between the $P_{Rn}$ and $O_{x}$ at time *t*, where $n\in \left\{C,V,H\right\}$ indicates the receiver. Finally, $i_{P_{T}}\left(t\right)$ is the LOS unit vector of the transmitted antenna, and $i_{P_{Rn}}\left(t\right)$ is the one for a receiving antenna.

As the distance between the system and the target is much larger than the distances separating the antenna, it is assumed that $a_{n}\approx a$ in the following.

Assuming that $i_{P_{n}}=i_{P_{T}}+i_{P_{Rn}}$. The scalar product $x\;\cdot \;i_{P_{n}}$ represents the distance between the focusing point $O_{x}$ and the projection of the scatterer onto the LOS. When the rotational vector is constant during the observation time Ω(t)≡Ω(0) for $\left|t\right|\leq T_{obs}$, the instantaneous displacement vector x(t) of the scatterer satisfies the differential equation:

(11) $$\frac{\partial x(t)}{\partial t}=\Omega \times x\left(t\right)$$

Using a first-order Taylor expansion for displacement, the received signal after motion compensation with range alignment is as follows:

(12) $$\begin{array}{c} \begin{array}{c} S_{n}\left(f,t\right)=a\exp\left(-j\frac{4\pi f}{c}\left[x_{0}\;\cdot \;i_{P_{n}}\right]\right)\mathrm{rect}\left(\frac{f-f_{o}}{B}\right)\\ \exp\left(-j\frac{4\pi f}{c}\left[\left(\Omega \times x_{0}\right)\;\cdot \;i_{P_{n}}\right]t\right)\mathrm{rect}\left(\frac{t}{T_{obs}}\right) \end{array} \end{array}$$

where $x_{0}=x\left(t\right)$ when t=0.

## 4. 3D Target Reconstruction

### 4.1. Estimation of the Positions of Scattering Centers

**Figure 5:**
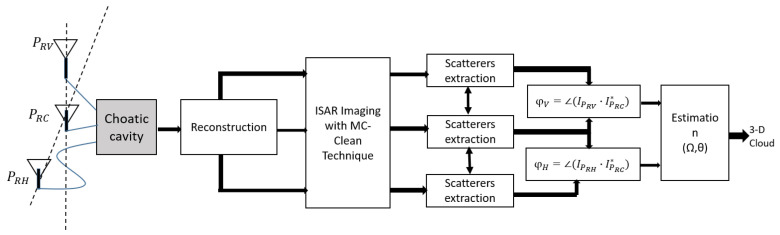
Block diagram of the dual baseline InISAR processing including a hardware compressive coding device.

The block diagram for the extraction and 3D localization of the scattering centers of the scene shown in Figure 5 is inspired by the original techniques proposed in [5,18]. Once the received signals have been estimated from the single output of the chaotic cavity, three ISAR images are first reconstructed with the conventional ISAR technique of [5,18].

The MC-Clean [5,18] aims to extract the brightest scattering center in one of the multichannel ISAR images and find its coordinates in the image plane. This algorithm performs an iterative process where the point spread function of the brightest scattering center is estimated and removed from the ISAR images. The algorithm stops when the residual energy in the ISAR image at the *k*th iteration is lower than a preset threshold, usually set a value significantly higher than the noise ground floor. The phase differences related to the actual elevation and Döppler rate of each scatterers, and to the location of each of the receiving antenna, are measured for the most dominant responses in the observed scene.

(13) $$\begin{array}{c} \begin{array}{c} \varphi_{V}=\frac{4\pi }{\lambda }\left(x_{0}\;\cdot \;\left(i_{P_{V}}-i_{P_{C}}\right)\right)\\ \varphi_{H}=\frac{4\pi }{\lambda }\left(x_{0}\;\cdot \;\left(i_{P_{H}}-i_{P_{C}}\right)\right). \end{array} \end{array}$$

The Doppler frequency is directly related to the cross-range coordinate, which may be expressed after some mathematical manipulations [36] as a function of the phases differences and the incidence angle θ:

(14) $$v_{c}=-\frac{d_{0_{c}}\Omega_{eff}}{2\pi }\left(\frac{\varphi_{H}}{d_{H}}\cos\left(\theta \right)+\frac{\varphi_{V}}{d_{V}}\sin\left(\theta \right)\right)$$

Now, the aspect angle θ and the effective rotation $\Omega_{eff}$ can both be estimated from (14) over a common linear regression plan. The components *z* represents the height of the scatterer with respect to the image projection plane, and can be approximated as a function of the phase differences and of the angle

(15) $$\begin{array}{c} \begin{array}{c} z\approx \frac{\lambda }{4\pi d_{0_{c}}}\left(\frac{\varphi_{V}}{d_{V}}\cos\theta -\frac{\varphi_{H}}{d_{H}}\sin\theta \right). \end{array} \end{array}$$

where λ is the wavelength and $d_{H}$ and $d_{V}$ stand for the effective horizontal and vertical baselines, respectively.

### 4.2. Baselines Constraints

The upper limits of the vertical and horizontal baselines that guarantee unambiguous phase measurements are given by:

(16) $$d_{V}⩽\frac{\lambda R_{0}}{2h_{V}},\;d_{H}⩽\frac{\lambda R_{0}}{2h_{H}}$$

where $h_{V}$ and $h_{H}$ are the maximum extent of the observed scatterers positions with respect to the imaging plane in both directions.

### 4.3. Image Distortion with Squint Mode

For a reconstruction of the InISAR 3D image in a cartesian reference frame $T_{1}$, a correction of the slanted and squinted geometry of the radar acquisition is necessary [37,38]. For a target located at t=0 at the position $\left({\hat{X}}_{o_{x}},{\hat{Y}}_{o_{x}},{\hat{Z}}_{o_{x}}\right)$ in the frame $T_{0}$, the orientations angles can be expressed as

(17) $$\begin{array}{c} \begin{array}{c} \alpha =\arctan\left(\frac{{\hat{X}}_{o_{x}}}{{\hat{Y}}_{o_{x}}}\right)\\ \beta =\arctan\left(\frac{{\hat{Z}}_{o_{x}}}{\sqrt{{\hat{X}}_{o_{x}}^{2}+{\hat{Y}}_{o_{x}}^{2}}}\right) \end{array} \end{array}$$

Then, the LOS direction for the system can be written as:

$$i_{LOS}=\left[\sin\beta \sin\alpha ,\sin\beta \cos\alpha ,\cos\beta \right]$$

For any scattering center *P* of the target, the projection coordinate of a point P onto the reference system $T_{1}$ is:

(18) $$r=O_{x}P\;\cdot \;i_{LOS}=\left|\begin{array}{c} x\sin\beta \sin\alpha \\ y\sin\beta \cos\alpha \\ z\cos\beta \end{array}\right|$$

Distortions of the original image geometry are due to the fact that the coordinates P are defined in the slant range, as shown in (18). Setting β=π/2 and α=0, transforms the slant range axis *R* into the ground the range axis *Y*, which corresponds to the standard reference configuration.

## 5. Simulation Results

**Figure 6:**
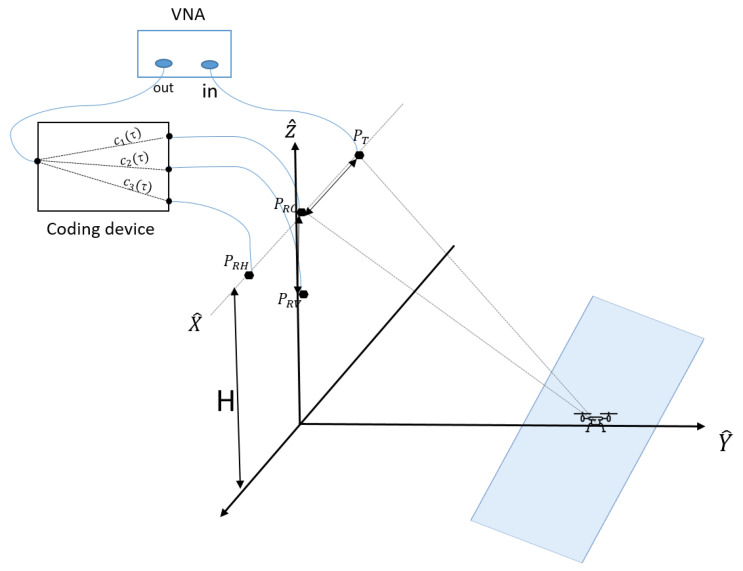
Simulated configuration including a hardware coding device connection and an antenna array.

**Figure 7:**
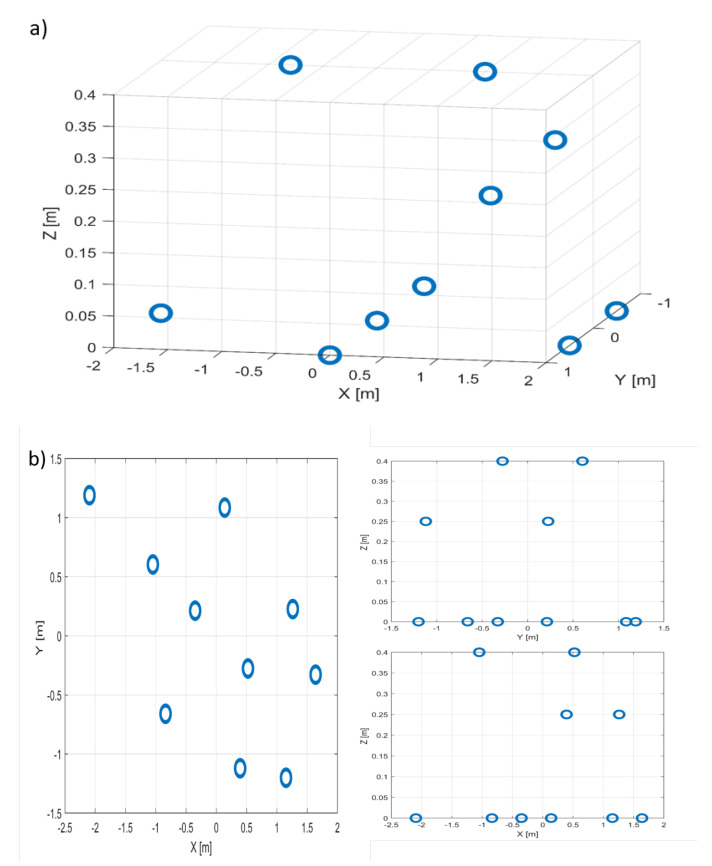
Simulated objet description. (**a**) 3D. (**b**) Top, side and front views.

The proposed approach is validated using a simulation performed according to the configuration shown in Figure 6. A rigid body mimicking the shape of an airplane composed of M=10 metallic scatterers is considered. The target moves on a straight line, defined by roll, pitch and yaw angles, with respect to the radar Line Of Sight (LOS), with a speed of 8 m/s. The parameters used numerically are shown in Table 1. The geometry of the target in X-Y-Z cartesian coordinates is displayed in Figure 7.

**Figure 8:**
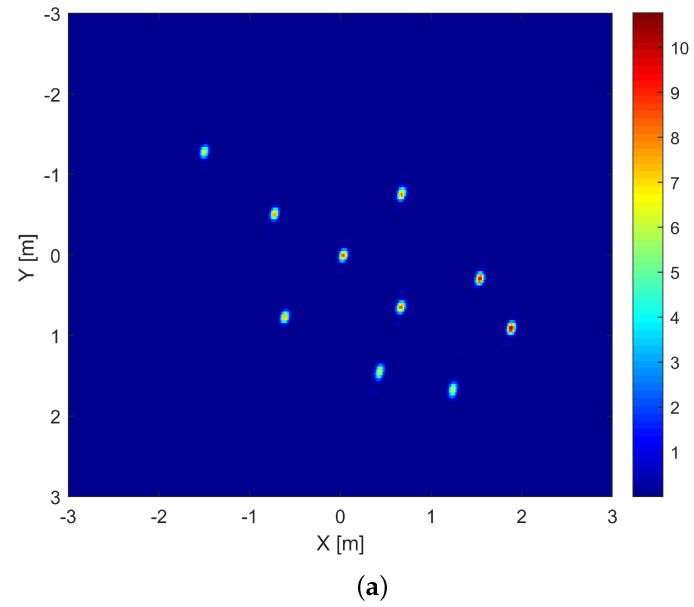

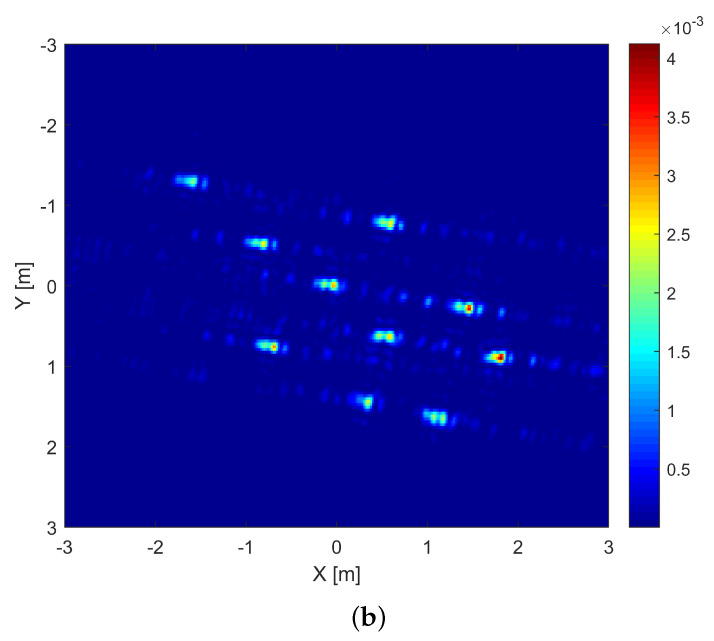
Two—dimensional images of a single channel after motion compensation of the simulated scene response. (**a**) measured by an antenna; (**b**) reconstructed from the output signal of the chaotic cavity.

**Figure 9:**
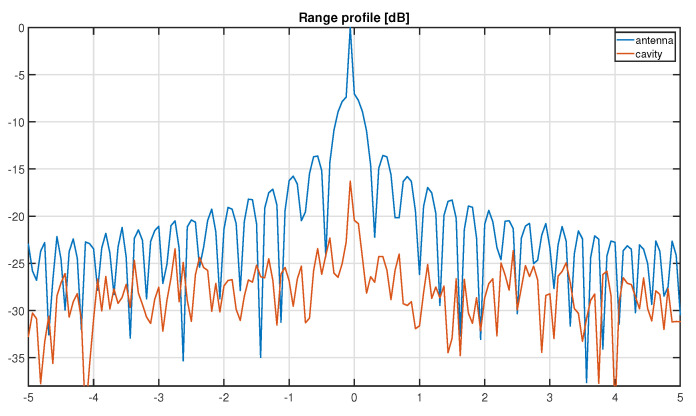
Normalized azimuth slice of the ISAR image of a single scatterer response.

The simulation of the response of the imaged set of scatterers is performed using advanced inverse techniques [39,40] in the case of a classical multi-channel system. Hardware compressed signals are then obtained by applying the chaotic cavity transfer function, measured experimentally, onto the simulated signals. Focusing results obtained using the Range–Doppler algorithm are shown in Figure 8, and indicate that the 2D imaging resolution is not deteriorated by the use of the hardware multiplexer. However, strong side lobes appear when the compressive device is used, as illustrated in Figure 9. These side lobes are inherent of the inversion procedure [31] and obviously limit the dynamic range of the reconstructed image.

**Figure 10:**
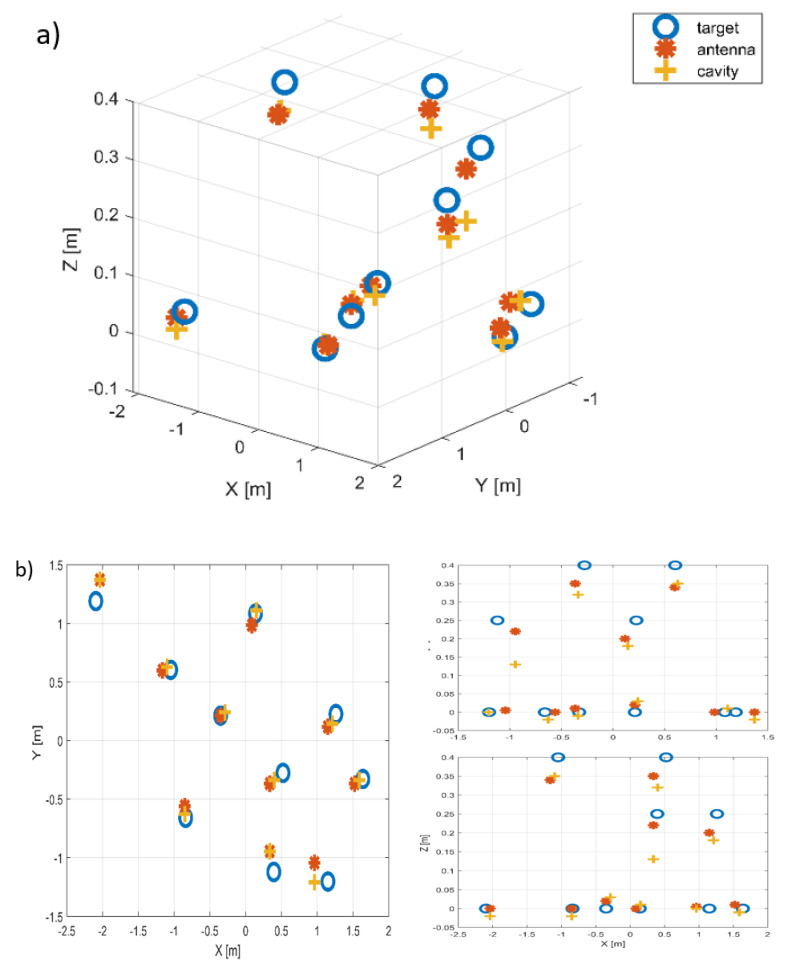
Reconstruction result: (**a**) 3D, (**b**) X-Y, X-Z and Y-Z planes. Blue circles represent true scatterer locations, red stars indicate those estimated using a multi-channel system, and yellow crosses represent results obtained using the chaotic cavity.

The 3D reconstruction of the scattering center position obtained with the Clean Technique is shown in Figure 10. The application of MC-Clean allows us to limit the influence of sidelobes induced by the reconstruction of the signals from the output of the chaotic cavity. Slight offsets on the location of the scatterers are, however, found in both cases, due to the approximations associated with the compensation of 2D uniform target rotation. The reconstruction is slightly deteriorated in the case of the compressive device, as the InSAR algorithm is sensitive to a reduced signal-to-noise ratio, as demonstrated in [5].

## 6. Experimental Result

An experiment was carried out using hardware and software facilities developed for 3D Ground-Based SAR campaigns dedicated to the refined characterization of volumetric environments [41,42,43,44]. The geometrical configuration was similar to the one used to simulate signals, but with a reference antenna $P_{C}$ located at a height of 5.5 m above the ground, and at ground range distance of 5.5 m from the center of the scene. The target composed of three trihedral reflectors—see Table 2—translated on a 3 m-long rail lying on the ground, with a step length of λ/4∼9 mm with an orientation of $60^{\circ }$. Measurements were first performed with a multi-port VNA connected to the antenna system, and then using the chaotic cavity and a single port.

**Figure 11:**
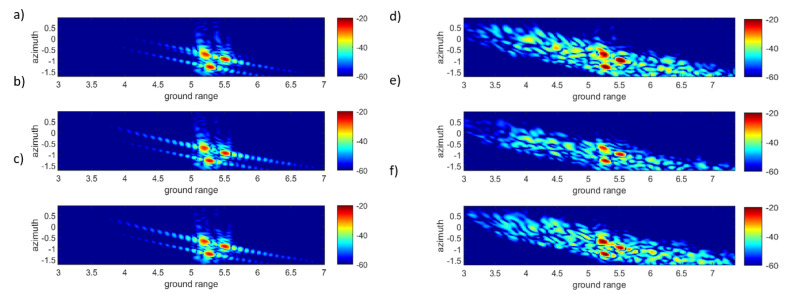
2—D images normalized obtained after motion compensation the signals on the three receivers antennas: measurement with antennas (**a**) $P_{C}$. (**b**) $P_{H}$. (**c**) $P_{V}$, with cavity after reconstruction: (**d**) $P_{C}$. (**e**) $P_{H}$. (**f**) $P_{V}$.

After compensation of the target trajectory, the RD algorithm was applied to the data measured by each antenna and after reconstruction with the compressive device. The ISAR images in range and cross-range coordinates are shown in Figure 11 and confirm that the use of a chaotic compressive hardware device induce stronger side lobes and affects the quality of the ISAR image. In this context, the Clean technique, which is able to extract the complete impulse response from the total signal, is very important, as high sidelobes may mask low-reflectivity scatterers.

**Figure 12:**
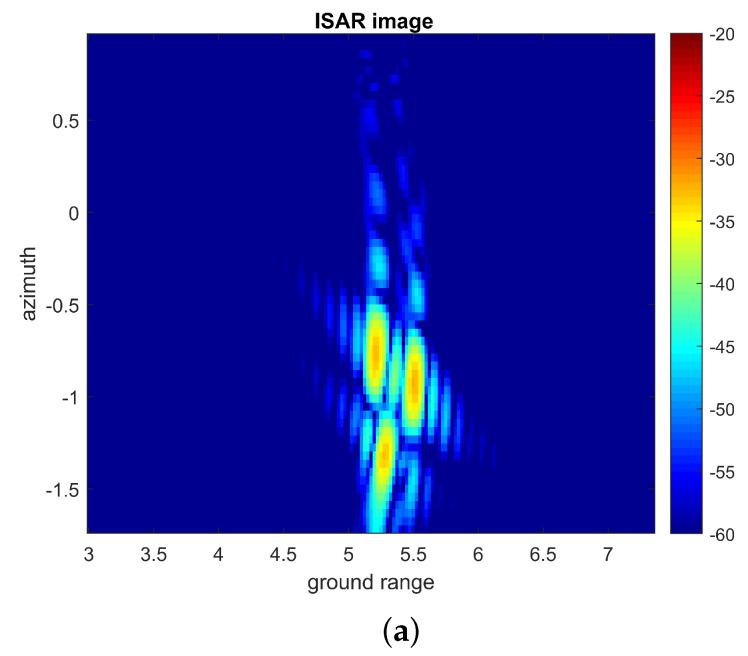

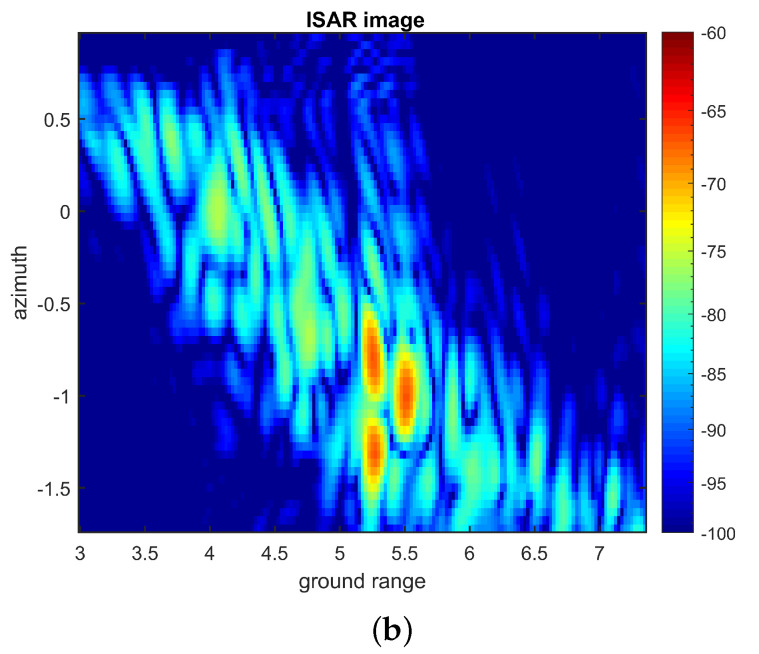
Two-dimensional images after motion compensation: (**a**) measurement with antennas $P_{C}$. (**b**) with cavity after reconstruction.

**Figure 13:**
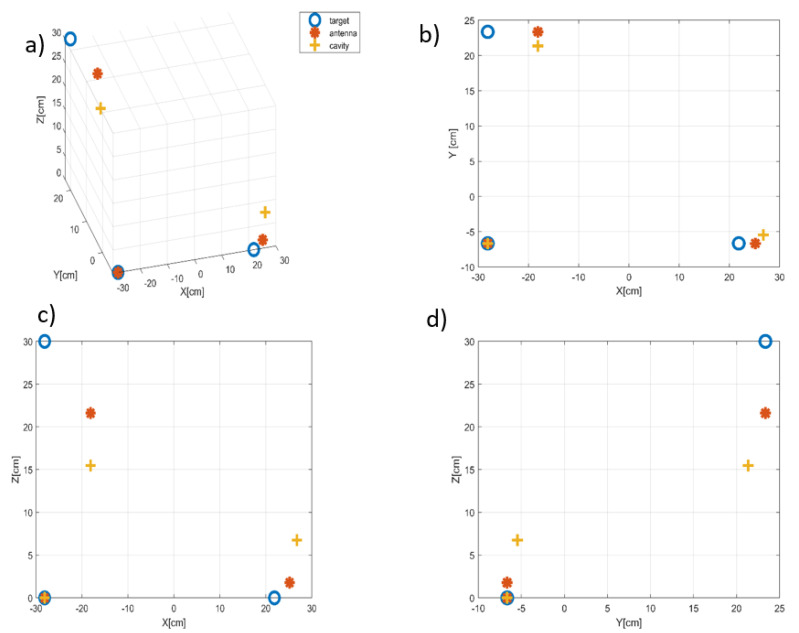
Scatterers positions retrieved using experimental data acquired with and without the cavity. (**a**) 3D, (**b**) top, (**c**) side, (**d**) front views.

Among a full dataset, a subset of data corresponding to the third of path was chosen for analysis (Figure 12), as it appears to limit migration in distance. Therefore, each scatterer must have the same range-azimuth coordinates for all three images. The MC-Clean technique was applied to the data with a threshold of 10% from the strongest peak image in the ISAR images for both measurements. A total of three scatterers was extracted for each measurement. The Figure 13 shows the estimated 3D cloud point in red for MC-clean with antennas, in yellow for MC-Clean after reconstruction, aligned with the model in blue.

The reconstruction operated using multiple receiving channels led to an RMS error of ζ=9 cm, whereas the one obtained with the compressive device was ζ=17 cm. Estimated target dimensions, whose calculation is a key aspect of InISAR, are given in Table 3. These results are in agreement with the actual target values. However, there are several factors that influence the results on the real data, such as the estimated position of the scene center and the phase difference between the antennas. A perfect trajectory compensation allows us to limit the errors of the Doppler measurement. The residual correlation between the transfer functions of the chaotic cavity may be considered as a source of error, too, as it affects the quality of phase calculation and Doppler measurement.

## 7. Conclusions

This paper proposes using a compressive device to reduce the complexity of an InISAR technique, which requires us to simultaneously measure signals at different antenna positions. The objective is to minimize the complexity of the acquisition system and still benefit from the robustness and simplicity of the InISAR 3D-focusing approach for a scaled reconstruction of a complex moving target. Simulations show that the hardware compressive device considered in this study, a chaotic cavity, does not affect the imaging resolution, but involves the presence of side lobes which may affect the characterization of complex targets. Nevertheless, the iterative estimation approach used in the InISAR method reaches good performance, as it subtracts the entire impulse of a scatterer from the total signal, i.e., it can handle sidelobes efficiently. Experimental results, obtained with a three-element L-shaped antenna array connected to a metallic enclosure, led to a reconstruction in very good agreement with true scatterer positions. Hence, this study demonstrates that compressive approaches represent a promising and low-cost solution to overcome synchronisation problems for acquisition of configurations involving several receiving channels.

## Figures and Tables

**Table 1:** Radar acquisition parameters.

Parameters	Values
Carrier frequency	6 GHz
Signal bandwidth	2 GHz
Baseline ($d_{h}=d_{v}$)	30 cm
Number of frequency samples	2001
Number of radar sweeps	120
PRF	800 Hz
Ground range	25.5 m
Roll/Pitch/Yaw	$0^{\circ }$ $0^{\circ }$ $60^{\circ }$
Radar height	5 m

**Table 2:** Trihedral reflector positions with respect to the target center.

Trihedral Number	(X, Y, Z)
1	(0, 0, 0)
2	(0.5, 0, 0)
3	(0, 0.3, 0.35)

**Table 3:** True and estimated target dimension.

Measurement	Length (cm)	Width (cm)	Height (cm)
True value	50	30	35
Multichannel InISAR	52	29	21
Single compressed channel	55	28	15

## Data Availability

Not applicable.
